# B Chromosomes in the *Drosophila* Genus

**DOI:** 10.3390/genes9100470

**Published:** 2018-09-27

**Authors:** Stacey L. Hanlon, R. Scott Hawley

**Affiliations:** 1 Stowers Institute for Medical Research, Kansas City, MO 64110, USA; rsh@stowers.org; 2 Department of Molecular and Integrative Physiology, University of Kansas Medical Center, Kansas City, KS 66160, USA

**Keywords:** *Drosophila*, supernumerary, satellite DNA, sSMC

## Abstract

Our current knowledge of B chromosome biology has been augmented by an increase in the number and diversity of species observed to carry B chromosomes as well as the use of next-generation sequencing for B chromosome genomic analysis. Within the genus *Drosophila*, B chromosomes have been observed in a handful of species, but recently they were discovered in a single laboratory stock of *Drosophila melanogaster*. In this paper, we review the B chromosomes that have been identified within the *Drosophila* genus and pay special attention to those recently found in *D. melanogaster*. These newly-discovered B chromosomes have centromeres, telomeres, and a number of simple satellite repeats. They also appear to be entirely heterochromatic since next-generation sequencing of isolated B chromosomes did not detect sequences associated with known genic regions. We also summarize what effects the B chromosomes have been found to have on the A chromosomes. Lastly, we highlight some of the outstanding questions regarding B chromosome biology and discuss how studying B chromosomes in *Drosophila melanogaster*, which is a versatile model system with a wealth of genetic and genomic tools, may advance our understanding of the B chromosome’s unique biology.

## 1. Introduction

All members of a single species carry a defined set of essential chromosomes or A chromosomes that are required for the normal growth, development, and reproduction of an organism. In many species, however, a subset of individuals can harbor nonessential, supernumerary chromosomes referred to as B chromosomes. First described by Wilson over a century ago, they have since been identified in hundreds of species spanning many different taxa [1,2,3,4]. Our understanding of B chromosome biology is currently undergoing rapid change due to the growing accessibility of advanced molecular and genomic techniques [5,6,7]. These modern methods are illuminating facets of B chromosome structure and biology that would not have been uncovered through classical cytological approaches such as the presence of active genetic material or the landscape of repetitive elements at a high resolution [8,9,10].

Since more B chromosomes are molecularly analyzed, an important next step will be to connect their genetic composition to known attributes. For example, several B chromosomes are subject to drive mechanisms that can result in their accumulation within a population, but, in some instances, the details of how those systems work have not been elucidated [11]. When the plant or animal systems carrying these B chromosomes are not conducive to experimentation in a laboratory setting, it can be difficult to definitively connect B chromosome genotypes to observed phenotypes.

**Figure 1:**
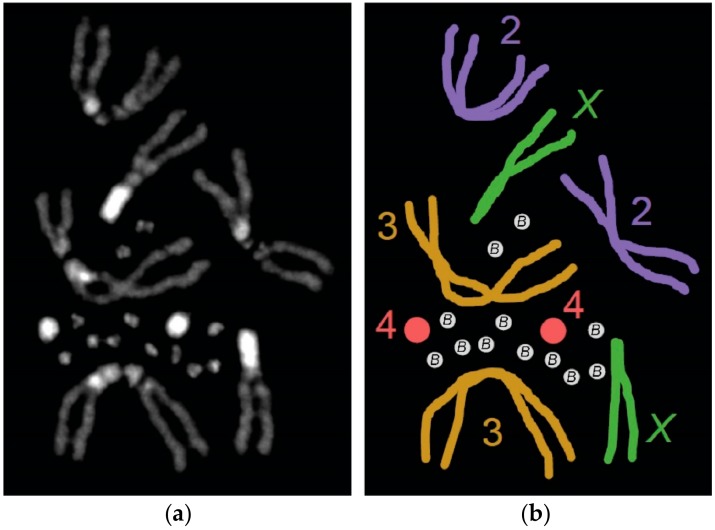
The B chromosomes of *Drosophila melanogaster*. (**a**) A karyotype from a female carrying 12 supernumerary B chromosomes. The DNA is stained with DAPI (photo by authors). (**b**) Illustrated representation of the karyotype in (**a**). Homologous chromosomes are shown in the same color.

Recently, B chromosomes were discovered in a laboratory stock of the robust model organism known as *Drosophila melanogaster* (Figure 1) [12]. These B chromosomes have since undergone a molecular analysis that is beginning to provide insight into their origin, composition, and structure [13]. In this review, we summarize the B chromosomes that have been observed in various species within the genus *Drosophila*, which was followed by an analysis of the emerging B chromosome model system in *D. melanogaster*. Lastly, we highlight some outstanding questions in B chromosome biology that may be well-suited for study in this emerging B chromosome system.

## 2. B Chromosomes within the Genus *Drosophila*

Out of over 1600 documented species within the *Drosophila* genus, more than 600 of these have been sampled to determine the shape and number of their chromosomes [14,15]. To date, supernumerary B chromosomes have been positively identified in just nine *Drosophila* species. The initial report documenting the existence of a B chromosome in each of these species is presented in Table 1. Since B chromosomes are generally small and can be easily overlooked in a karyotype and, because within the same species they can be present in some populations and absent in others, this low frequency of B chromosomes in *Drosophila* is likely an underestimate. 

The first reported B chromosome in *Drosophila* was identified by two separate groups who collected independent samples of *D. albomicans* (also referred to as *D. nasuta albomicana*) from wild populations around Chiang Mai, Thailand [16,17]. Clyde collected individual females to create isolines that were each examined cytologically. Three out of eight isolines carried supernumerary “dot” chromosomes in addition to the normal karyotype and individuals within the same isoline could carry up to two copies [16]. The second strain of *D. albomicans* from Thailand was cultured in the laboratory of Osamu Kitagawa [17]. Ramachandra and Ranganath cytologically analyzed this strain and determined that individuals were able to carry one, two, or three copies of the B chromosome [23]. In subsequent studies, they were also able to document the rapid accumulation of B chromosomes within the strain after being kept under laboratory conditions [24]. In just three years, the percentage of individuals carrying at least one B chromosome rose from 67% to 98% and the frequency of individuals carrying three or more B chromosomes jumped from 5% to 40% [25]. The B chromosome of *D. albomicans* likely possesses the ability to promote its rapid propagation within a population since samples collected from diverse locations in Southeast Asia have been found to carry B chromosomes [26]. Though an analysis of heterochromatic composition through various staining techniques revealed that the *D. albomicans* B chromosomes differ from the A chromosomes, no conclusions were drawn regarding how those differences may influence the proliferation of the B chromosome [27]. Perhaps a closer examination of the recent genomic sequence of *D. albomicans* will provide the key [28].

**Figure 2:**
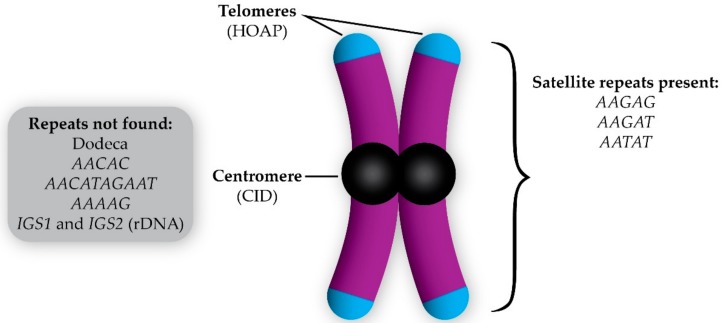
Composition of the *D. melanogaster* B chromosome (initially described in Reference [12]). The B chromosome has telomeres (indicated by the presence of HOAP, which is a telomere capping protein), centromeres (due to the incorporation of the centromeric histone CID), and a variety of satellite repeats revealed by fluorescent in situ hybridization. The repetitive sequences not found on the B chromosomes are listed in the gray box.

The second *Drosophila* species reported to carry a B chromosome was also from Southeast Asia. Samples of *D. malerkotliana* collected throughout the region—from Mauritius and Seychelles to all along the coast of India, Myanmar (Burma), Thailand, and Malaysia—were shown cytologically to carry B chromosomes [18]. Another species collected from India, *D. kikkawai*, carries two distinct B chromosome variants in which each is considerably larger than the B chromosomes from *D. albomicans* and *D. malerkotliana* [19]. When these stocks were selected for the presence of B chromosomes in a laboratory setting, the maximum copy number of the B chromosomes we were able to reach was four [29]. Even though this is the only report of B chromosomes in *D. kikkawai*, it would be interesting to re-examine early cytological data while keeping the possibility of an unidentified B chromosome in mind. For example, in a different sample of *D. kikkawai*, there appears to be a darkly-staining fragment present in one of the mitotic spreads (see Figure 2B in Reference [30]). Since this is the only image taken of the sample, it is not known if other metaphases from the same sample also had this fragment, which makes it impossible to determine if the fragment is a B chromosome or simply an artifact of the experiment. A similar oversight in *D. flavopilosa* may have occurred in 1962: Bencic briefly mentions “an extra dot-like chromosome” that is not present in all samples, which very well may have been a B chromosome [31].

B chromosomes have also been found in *Drosophila* specimens collected outside of Southeast Asia. Samples of *D. subsilvestris* collected on two separate occasions from Southwest Germany each carried B chromosomes [20]. The first of the established lines had up to five dot-like chromosomes shortly after its collection, but after ~14 years in the lab, the dot-like chromosomes were all but lost. The second line was collected from the wild and established when the first line died out. This new stock also carried dot-like chromosomes and they were soon determined to be B chromosomes since they did not appear to undergo polytenization, which indicates that they were heterochromatic. Additionally, the B chromosomes carry the heterochromatic satellite DNA repeat *pSsP216*, which indicates that they may have arisen from the dot chromosome that also carries this repeat [20].

A large cytological survey of 34 species from the *melanogaster* species group revealed that *D. lini* and *D. pseudoananassae* collected from China can have extra chromosomes that are potential B chromosomes [21]. The size of the *D. lini* supernumerary chromosomes—as well as the consistency of the A chromosome karyotype—suggests that these are likely to be B chromosomes. The extreme range of karyotypic variations observed in *D. pseudoananassae*, however, confounds the assessment of whether some of the chromosomes are truly supernumerary and nonessential and a closer examination of this species is required. More cytological studies are also necessary in *D. yangana* and *D. huancavilcae*, which reportedly have supernumerary chromosomes but have not been cytologically documented in the literature [22].

Recently, B chromosomes were detected in *D. melanogaster*, which is the best-studied species of *Drosophila* [12]. As a widely used model organism with an extensive history of laboratory experimentation, *D. melanogaster* has a wealth of genetic and molecular tools that can be utilized to understand important aspects of B chromosome biology. Some of these aspects are discussed below.

## 3. B Chromosomes in *Drosophila melanogaster*

Two studies documenting and molecularly characterizing the B chromosomes in *D. melanogaster* were recently conducted in the Hawley laboratory [12,13]. The findings of the initial study [12] and emerging results from a second study [13] are summarized below.

### 3.1. Characterization of the Original Drosophila melanogaster B chromosome

The first B chromosomes identified in *D. melanogaster* were discovered in a Hawley laboratory stock that carries a null mutation in *matrimony*, which is a Polo-kinase inhibitor that acts only during female meiosis in *Drosophila* [32,33,34]. While examining female oocytes during meiotic prometaphase I in this mutant, DAPI-staining fragments that were much smaller than chromosome *4* were observed along the meiotic spindle. Initially, these were assumed to be the common *Drosophila* intercellular bacterial parasite, *Wolbachia*. This presumption was challenged when immunofluorescence (IF) using antibodies recognizing the *Drosophila* centromeric histone Centromeric Identifier (CID) revealed these fragments incorporated CID [12]. IF also revealed that these fragments were coated with H3K9 methylation, which is an epigenetic mark of silenced chromatin. This indicated that they were mostly heterochromatic [12]. The presence of centromeres signified these fragments were chromosomes and, since they were small, heterochromatic and, when they carried in addition to the full complement of A chromosomes, they were classified as supernumerary B chromosomes [12]. Additionally, it appears that these fragments may also have telomeres as indicated by the presence of HOAP (encoded by *cav*), which is a telomere-specific capping protein in *Drosophila* (Figure 2) [13].

The first step toward finding the origin of the *D. melanogaster* B chromosomes was to determine their composition. Fluorescent in situ hybridization (FISH) using probes for a variety of repetitive sequences has revealed that the B chromosomes carry satellite DNA sequences that are also found on the A chromosomes such as the *AATAT* (chromosomes *4*, *X*, and *Y*) and *AAGAG* (all chromosomes) repeats [12,13]. The repeat sequences from the A chromosomes that were not found on the B chromosomes were Dodeca (chromosome *3*), *AACAC* (chromosomes *2* and *Y*), *AACATAGAAT* (chromosomes *2* and *3*), *AAAAG* (chromosomes *2* and *Y*), and the intergenic spacer (IGS) that is between ribosomal DNA repeats (chromosomes *X* and *Y*) [12,13]. Of the repeats tested, the most prominent satellite repeat on the B chromosomes as assayed by FISH appears to be the *AAGAT* repeat (Figure 2). The only A chromosome that also carries this repeat is chromosome *4*, which is consistent with the hypothesis that the B chromosomes originated from chromosome *4* [13]. 

Chromosome *4* in *D. melanogaster* has euchromatin near the tip of its right arm and the remainder of the chromosome is heterochromatin. Even though the B chromosomes appear to be mostly heterochromatic (based on H3K9 methylation), they may carry part of the euchromatic tip from chromosome *4*. Thus, they were initially assayed for the presence of several genes present in this euchromatic region both genetically via complementation tests as well as molecularly through a qPCR-based analysis. Both approaches indicated the B chromosomes do not carry chromosome *4* genes [12].

To conduct a deeper molecular examination of their composition, the B chromosomes were separated from the A chromosomes via pulsed-field gel electrophoresis (PFGE) [13]. Using this method, the B chromosomes appear to be homogeneous in size at approximately 1.8 Mbp. The DNA band corresponding to the B chromosomes was extracted from the gel and subjected to next-generation sequence analysis in the absence of the A chromosomes. Even though no known genic regions were detected, a number of genetic elements most often associated with heterochromatin (e.g., transposons and satellite repeat sequences) were found in which a large portion was full of repetitive sequences also found on chromosome *4* [13].

### 3.2. Effects of the Drosophila melanogaster B Chromosomes on the A Chromosomes

Stocks that carry an average of 10 to 12 B chromosomes do not have an obvious phenotype. However, the presence of B chromosomes can have a measurable effect on the A chromosomes. During female meiosis, the segregation of chromosome *4* is disrupted when B chromosomes are present, which increases their frequency of mis-segregation by nearly two orders of magnitude [12]. The mechanism of this disruption is presently unknown. The achiasmate (non-crossover) system that segregates chromosome *4* may be encumbered in the presence of B chromosomes or possibly due to the similarity of heterochromatic sequence between the B chromosomes and chromosome *4* is affecting the establishment of heterochromatin associations earlier in meiosis [35,36,37].

The B chromosomes can also influence position effect variegation (PEV), which occurs when a gene in a euchromatic region is intermittently silenced due to its proximity to a heterochromatic region [38]. Placing the visual genetic marker *white* (*w*), which controls the degree of eye pigmentation, into one of these regions will lead to eyes with a variegated color pattern. Though the mechanism of PEV suppression is not entirely understood, it is thought that the presence of excess heterochromatin (such as an extra *Y* chromosome or an extra chromosome *4*) has the ability to act as a sink for silencing proteins. This would reduce the overall spread of heterochromatin into nearby euchromatin, which increases the expression from *w* to the result in eyes with more pigment and less variegation. The B chromosomes were shown to affect PEV at multiple regions in the genome, which indicates that their presence can alter the heterochromatic silencing boundaries of the A chromosomes [12].

## 4. The *D. melanogaster* B Chromosome as an Emerging Model for B Chromosome Biology

Every B chromosome has its own unique biology and there is no shortage of interesting questions that can be asked. Two recurring questions—how are B chromosomes formed and what is the mechanism of B chromosome maintenance over time—are challenging to answer in non-model systems that lack genetic and molecular tools. The B chromosomes of *D. melanogaster* are poised to be a powerful system to begin studying how B chromosomes arise as well as what is required for them to be maintained and proliferate through a stock.

Currently, cytological and molecular analysis of the *D. melanogaster* B chromosomes indicates they arose from chromosome *4*, but the underlying mechanism of their creation is unknown. Examination of the *AAGAT* FISH signal shows that it is more abundant on the B chromosomes, which leads to the hypothesis that the B chromosomes formed after a centromere mis-division of chromosome *4* during meiosis [13]. To test this hypothesis, a chromosome with markers on either end would need to be created and monitored after hundreds—or possibly thousands—of meiotic events. With the genetic toolbox available in *D. melanogaster*, marking a specific site within the genome with a visible genetic marker is a routine task. Additionally, the husbandry of *D. melanogaster* in the laboratory is easily amenable to large-volume cultures, which enables the researcher to screen as many flies as necessary. It is also unclear whether the appearance of the B chromosomes was fortuitous or if the genotype of the stock played a role in their creation. Once a basal frequency of B chromosome formation can be established, this question can be addressed by testing whether the *matrimony* genetic background influences the frequency of B chromosome formation. From here, one can test a variety of other existing mutant *D. melanogaster* stocks that are curated in the Bloomington *Drosophila* Stock Center and available to the research community.

How the original *D. melanogaster* B chromosomes have been maintained in such high copy number for several years is also puzzling. B chromosomes in other systems have been shown to promote their own propagation to the next generation through a variety of drive mechanisms (for a review, see “Transmission and Drive Involving Parasitic B Chromosomes” that is included in this special issue [11]). It is clear that the *D. melanogaster* B chromosomes do not supply their own mechanism of drive [12] and no known genes were detected after their genomic analysis [13]. Therefore, one hypothesis is that the genetic background of the stock may be responsible for their maintenance. It is known that *matrimony* plays an essential role in *Drosophila* female meiosis and is required for the accurate segregation of chromosomes that do not form crossovers [32,33,34]. Since *D. melanogaster* B chromosomes do not appear to form crossovers (as demonstrated by their dynamics on the meiotic spindle [12]), the disruption of *matrimony* may be influencing their segregation. Observing how the B chromosomes segregate is attainable in *D. melanogaster* either in fixed specimens using established protocols or in live egg chambers with a menagerie of fluorescent tools [39,40]. Ultimately, studying B chromosome formation and behavior in such an established model system will accelerate our understanding of general B chromosome biology, which may be applicable to other systems that are less amenable to laboratory research.

## 5. Conclusions

The first *Drosophila* species reported to carry B chromosomes was *D. albomicans* from Thailand over 30 years ago. Since then, very few *Drosophila* species have been found to carry B chromosomes, but it is fortunate that one of them is *D. melanogaster*. With its powerful set of genetic and genomic tools, there is much to learn about the *D. melanogaster* B chromosomes. Dissecting the biology of B chromosomes in such a robust and versatile model system will provide insight into other B chromosome systems as well as small supernumerary chromosomes (sSMC) found in humans that can be detrimental to our health. These sSMC are similar to B chromosomes in many ways and have been associated with various syndromes, intellectual disabilities, and infertility [41,42,43]. Thus, the B chromosomes in *D. melanogaster* stand to be a promising model system with discoveries that will potentially be of value to both biology and medicine.

## Figures and Tables

**Table 1:** Initial reports of B chromosomes in different species of *Drosophila.*

Year Reported	*Drosophila* Species	Location of Sample Collection	Reference
1980	*D. albomicans* ^1^	Chiang Mai, Thailand	[16,17]
1983	*D. malerkotliana*	Multiple locations in Southeast Asia	[18]
1994	*D. kikkawai*	Bhubaneswar, India	[19]
1995	*D. subsilvestris*	Tübingen, Germany	[20]
2007	*D. lini*	Malipo county, Yunnan province, China	[21]
2007	*D. pseudoananassae*	Jianfengling, Hainan province, China	[21]
2009	*D. yangana* ^2^	Loja province, Ecuador	[22]
2009	*D. huancavilcae* ^2^	Manabí province, Ecuador	[22]
2014	*D. melanogaster*	Domesticated laboratory stock	[12]

^1^ Also referred to as *D. nasuta albomicana*. ^2^ B chromosomes reported but not cytologically recorded.
